# Receptor-Targeting Phthalocyanine Photosensitizer for Improving Antitumor Photocytotoxicity

**DOI:** 10.1371/journal.pone.0037051

**Published:** 2012-05-31

**Authors:** Peng Xu, Jincan Chen, Zhuo Chen, Shanyong Zhou, Ping Hu, Xueyuan Chen, Mingdong Huang

**Affiliations:** 1 Danish-Chinese Centre for Proteases and Cancer, State Key Laboratory of Structural Chemistry, Fujian Institute of Research on the Structure of Matter, Chinese Academy of Sciences, Fuzhou, Fujian, China; 2 Graduate University of Chinese Academy of Sciences, Shijingshan District, Beijing, China; 3 Key Laboratory of Optoelectronic Materials Chemistry and Physics, Fujian Institute of Research on the Structure of Matter, Chinese Academy of Sciences, Fuzhou, Fujian, China; Instituto de Investigación Sanitaria INCLIVA, Spain

## Abstract

Photodynamic therapy (PDT) is a promising therapeutic modality which uses a photosensitizer to capture visible light resulting in phototoxicity in the irradiated region. PDT has been used in a number of pathological indications, including tumor. A key desirable feature of the photosensitizer is the high phototoxicity on tumor cells but not on normal cells. In this study, we conjugate a gonadotropin-releasing hormone (GnRH) to a photosensitizer, Zinc phthalocyanine (ZnPc), in order to enhance its specificity to breast cancer, which over-expresses GnRH receptor. ZnPc has unique advantages over other photosensitizers, but is difficult to derivatize and purify as a single isomer. We previously developed a straight-forward way to synthesize mono-substituted β-carboxy-phthalocyanine zinc (ZnPc-COOH). Photophysical and photochemical parameters of this ZnPc-GnRH conjugate including fluorescence quantum yield (Ф_f_), fluorescence decay time (τ_s_) and singlet oxygen quantum yield (Ф_Δ_) were evaluated and found comparable with that of ZnPc, indicating that addition of a GnRH peptide does not significantly alter the generation of singlet oxygen from ZnPc. Cellular uptakes and phototoxicities of this conjugate were tested and found significantly enhanced on human breast cancer cell lines overexpressing GnRH receptors (MDA-MB-231 and MCF-7 cells) compared to cells with low levels of GnRH receptors, such as human embryonic lung fibroblast (HELF) and human liver carcinoma (HepG2) cells. In addition, the cellular uptake of this conjugate toward MCF-7 cells were found clearly alleviated by a GnRH receptor blocker Cetrorelix, suggesting that the cellular uptake of this conjugate was GnRH receptor-mediated. Put together, these findings revealed that coupling ZnPc with GnRH analogue was an effective way to improve the selectivity of ZnPc towards tumors with over-expressed GnRH receptors.

## Introduction

Cancer is a leading cause of death and a major public health problem worldwide. A WHO report on global cancer in 2008 pointed out that more than 70% of all cancer deaths occurred in low- and middle-income countries [1]. Deaths from cancer worldwide are projected to continue to rise to over 11 million in 2030 [1]. Traditional cancer treatments, including surgery, radiation therapy and chemotherapy, can cause serious side effects resulted from the damage of normal cells around. Photodynamic therapy (PDT) is regarded as a new promising cancer treatment modality, which typically involves the intravenous injection of a photosensitizer and the illumination by a visible light with appropriate wavelength activating phototoxicity of the photosensitizer by generating reactive free radicals. The phototoxicity of the illumination can usually affect up to 1–2 cm in depth [2], [3]. The selectivity of PDT toward tumor, mainly achieved by the selective accumulation of these photosensitizers within tumor tissue and their activation after light exposure, is usually quite poor and becomes one major issue that limits the wider application of PDT as a treatment modality [4]. Many photosensitizers used in PDT nowadays have limited selectivity for malignant cells, and thus significant amount of photosensitizer can be uptaken by normal tissues including skin which maybe one of the reason leading to skin photosensitivity. New generations of photosensitizers with better tumor selectivity are under active development in recent years [5], [6], [7].

We previously described a peptide-conjugated photosensitizer (ZnPc-(Lys)_5_), zinc phthalocyanine (ZnPc) conjugated with pentalysine peptidyl moiety, and reported its *in vitro* and *in vivo* efficacy [8]. The pentalysine peptide renders water solubility of ZnPc that is otherwise not soluble in physiological condition and requires specialized formulation with, e.g., Cremophor EL. In addition, the positive charges of pentalysine carries under physiological condition may provide the selectivity towards tumor, which carries more negative charges on the cell surface due to its active metabolism compared to normal cells. Indeed, this photosensitizer showed 2- and 6-fold selectivity for tumor over muscle and brain tissues respectively on S180 tumor-bearing mice [8].

In this study, a gonadotropin-releasing hormone (GnRH), also referred to as LHRH (luteinizing hormone-releasing hormone), was used as receptor-targeting peptide. GnRH is a hypothalamic decapeptide with the sequence of EHWSYGLRPG and is responsible for the release of follicle-stimulating hormone and luteinizing hormone from the anterior pituitary via its specific G-protein coupled GnRH receptor [9]. Notably, GnRH receptors are found aberrantly expressed in sex steroid-dependent tumors including breast, ovarian, endometrial, and prostate tumors [10], [11]. In view of the abundance GnRH receptor on these tumors, targeted chemotherapy based on GnRH analogues has gained considerable attention. However, the GnRH analogs show only moderate inhibition of tumor grow (15–20% inhibition after three to four days of treatment), making them not particularly useful for the treatment of breast cancer [9], [12].

On the other hand, various cytotoxic compounds conjugated with GnRH analogues have been evaluated for their anticancer activities. They exhibited a wide range of specific binding affinities toward cell surface GnRH receptors and could also be internalized by the cells [13], [14], [15]. In addition, attaching GnRH analogues to magnetic nanoparticles, such as iron oxide [12], [16] and iron-platinum nanoparticles [17], were reported to be able to facilitate their accumulation in cancer cells by targeting the GnRH receptors. Unfortunately, these conjugates were found to have serious side effects including the damage to normal pituitary gonadotropes which is probably due to their high penetration through blood-brain barrier and concomitant binding to healthy cells. Although the damage to pituitary cells was reported to be reversible [18], the use of these conjugates for treatment of malignancies should be carefully considered.

Our previous work showed that ZnPc has a very low level of distribution in murine brain [8], thus the ligation of ZnPc to GnRH may reduce the penetration of the conjugate through blood-brain barrier, and minimize the side effect of GnRH in brain. Herein we report the synthesis and the physicochemical characterization of this ZnPc-GnRH conjugate, along with the evaluation of its photodynamic activity at cellular level. Our results clearly demonstrate the selective cellular uptake and cellular phototoxicity of ZnPc-GnRH conjugate that are mediated by the cell surface GnRH receptor. This work paves a road for future *in vivo* studies.

## Materials and Methods

### Synthesis and Purification of GnRH Conjugate

Mono-substituted β-carboxyphthalocyanine zinc (ZnPc-COOH, **5**) was synthesized first as previously described [19]. ZnPc-GnRH conjugate (**7**) was then prepared by coupling **5** with side chain protected GnRH peptide on Wang resin (as shown in **Scheme S1**). Typically, ZnPc-COOH (9.3 mg, 0.015 mmol) was dissolved in N’N-dimethylformamide (DMF, 1.5 ml) first and incubated with carboxyl activation agent hexafluorophosphate tetramethylurea (HBTU, 23.1 mg, 0.06 mmol) and a base N, N-Diisopropylethylamine (DIEA, 0.093 ml) for 30 min at room temperature. Side chain protected GnRH peptide (50 mg, 0.015 mmol) on Wang resin (GL Biochem Ltd., Shanghai, China) was added into the solution and the mixture was kept stirring over night at room temperature to allow the reaction to complete. After that, the Wang resin was washed with DMF followed by methanol. To cleave the coupled product from the Wang resin and to cleave the protecting groups from the side chain of GnRH, the resin was soaked in 95% trifluoroacetic acid (TFA) for 4 hr. Crude product **7** was then precipitated by adding dried ether and the solvent was stripped off under vacuum. The compound **7** was further purified on a preparative HPLC (Dalian Elite Analytical Instruments Co. Ltd., Dalian, China) using a SinoChrom ODS-BP column (250×20 mm, 10 µm), eluting with a linear gradient of 10 to 100% acetonitrile (in water solution) during a period of 25 min at a flow rate of 5 ml/min. The final product **7** has high purity of 99.3% but the yield after purification was low (17.4% based on compound **5**). The low yield is partly due to the lost during purification, but also probably reflecting the heterogeneity of the GnRH peptide synthesized on resin.

### Characterization of GnRH Conjugate

ZnPc-GnRH conjugate (**7**) was characterized by ESI-MS (DECAX-30000 LCQ Deca XP), ^1^H-NMR (Bruker AV-400, 400 MHz, [D_6_] DMSO) and FT-IR (Magna-IR 750, Nicolett, KBr). ESI-MS results (**Fig. S1**): found *m*/*z* [*M*+H]^+^  = 1806.1; calcd *m*/*z* for C_88_H_90_N_24_O_16_Zn [*M*+H]^+^  = 1806.2. ^1^H-NMR (**Fig. S2**, [D_6_] DMSO): δ  = 14.3 (s, 2H), 11.5 (s, 1H), 10.8 (s, 1H), 6.5–10 (m, 38H), 5.8 (s, 1H), 2.7–5.2 (m, 25H), 2.4–1.2 (m, 15H), 0.9 (m, 6H) ppm. FT-IR results (**Fig. S3**): υ = 3271 cm^–1^ (N-H stretch), 1653 cm^–1^ (amide I), 1530 cm^–1^ (amide II), 1202 cm^–1^ (COO^−^ stretch). In addition, UV/Vis absorption spectrum of ZnPc-GnRH conjugate in dimethyl sulfoxide (DMSO) was recorded from 400 to 800 nm using quartz cuvettes with 1 cm path length on a Lambda-35 UV/Vis spectrometer (PerkinElmer, Massachusetts, USA) [λ_max_(e)  = 684 nm, molar extinction coefficient: 312880 Lmol^−1^ cm^−1^)]. Analytical HPLC was carried out on a C18 RP HPLC system (Dalian Elite Analytical Instruments Co. Ltd., Dalian, China; column: SinoChrom ODS-BP, 250×4.6 mm, 5 µm) using a linear gradient of 10 to 100% acetonitrile at a flow rate of 1 ml/min.

### Photophysical Properties of GnRH Conjugate

Fluorescence quantum yield (Ф_f_) of ZnPc-GnRH conjugate was measured by a comparative method using ZnPc as a standard (Ф_f (ZnPc) in DMSO_  = 0.2) [20] using the Eq. (1)
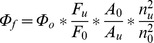
(1)where Ф_f_ and Ф_0_ are fluorescence quantum yields of the conjugate and standard, respectively. A_u_ and A_0_ are absorbances of the conjugate and standard, respectively. F_u_ and F_0_ are areas under the emission curves of the conjugate and standard, respectively. n_u_ and n_0_ are refractive indices of the solvents used for the conjugate and standard, respectively. The fluorescence of the conjugate and standard were measured on a Cary Eclipse Fluorescence spectrophotometer (Varian, Inc., Walnut Creek, CA).

Fluorescence decay time (τ_s_) of ZnPc-GnRH conjugate was measured by time-correlated single photon counting experiment of fluorescence excited with a 1.2 ns pulse laser diode at 460 nm, performed on an Edinburgh Instruments FLS920 spectrofluorometer (Livingston, UK) at 300 K. Fluorescence lifetimes were estimated by fitting the decay data using an interactive deconvolution procedure based on Marquardt algorithm with software supplied by OriginLab.

### Singlet Oxygen Quantum Yield Determination

The PDT effect of ZnPc type photosensitizer is usually mediated by type II mechanism involving singlet oxygen (^1^O_2_) as major cytotoxic agent. The quantum yield of ^1^O_2_ generation by ZnPc-GnRH was determined by comparing with the un-substituted zinc phthalocyanine (ZnPc) as reference [8], which has a quantum yield of 0.67 in DMSO [21]. Briefly, a 2 ml DMSO solution of the mixture of the ZnPc-GnRH conjugate (0.5 µM) and 1,3-diphenylisobenzofuran (DPBF, a scavenger molecule for ^1^O_2_), was introduced to the spectrophotometric cell of 1 cm path length and illuminated with a laser light (670 nm at 80 mW cm^−2^) in the presence of air (without degassing). The DPBF decay at 417 nm was monitored every 5 s. The concentration of DPBF was kept lower than 3×10^−5^ M during the experiment to avoid chain reactions induced by DPBF in the presence of ^1^O_2_. The quantum yield measurement has an error of less than 10%.

### Cell Lines and Culture Conditions

The cell lines we used in the current study were human breast cancer cell lines (MDA-MB-231 and MCF-7), human hepatoblastoma cell line (HepG2) and human embryo lung fibroblasts (HELF). All these cell lines were purchased from American Type Culture Collection (ATCC, Manassas, VA, USA).

MDA-MB-231 and MCF-7 cells were maintained routinely in Dulbecco’s modified Eagle’s medium (DMEM) supplemented with 10% fetal calf serum (FCS) and antibiotics. HepG2 and HELF cells were grown in RPMI-1640 supplemented with 10% FCS and antibiotics. All cells were kept at 37°C in a humidified incubator with 5% CO_2_ atmosphere. The viability of cells was determined by Trypan blue dye exclusion. Cells were maintained in logarithmic phase with viability >95%.

### Cellular Uptake of ZnPc-GnRH Conjugate

Aliquots of cells (2.5×10^5^ per ml growth medium) were placed in 96-multiwell black opaque plates (Costa) and incubated overnight at 37°C under 5% CO_2_. Various concentrations (10^−7^, 10^−6.5^, 10^−6^, 10^−5.5^, 10^−5^ and 10^−4.5^ M) of ZnPc-GnRH conjugate (in 1% DMSO) or ZnPc-COOH (in an additional 0.2% Cremophor EL) were added and incubated with cells at 37°C. After 12 hr incubation, the exponentially growing cells were washed with sterile PBS before lysis with NaOH (0.1 N, 0.2 ml with 1% SDS) to give a homogenous solution. The fluorescence of the cell extract was measured on a microplate reader (Synergy 4, BioTek Instruments) with λ_ex_  = 610 nm and λ_em_  = 690 nm. The concentration of cellular protein was determined with a BCA Protein Assay Kit (BioTeke Corporation, Beijing, China). Standard curves were made with cell lysates treated as above with known added amounts of bovine serum albumin. Results are expressed as nmol photosensitizer per mg cell protein.

### Uptake of ZnPc-GnRH Conjugate on MCF-7 in the Presence of a GnRH Antagonist

200 µl of MCF-7 cells (at a density of 2.5×10^5^ per ml) were seeded in each well of 96-multiwell black opaque plates and incubated overnight at 37°C under 5% CO_2_. After treated with or without Cetrorelix (at a concentration of 7.5×10^−7^ M), a GnRH antagonist, for 12 hr at 37°C, MCF-7 cells were washed twice with sterile PBS before fresh medium was added. These exponentially growing MCF-7 cells were then allowed to incubate with ZnPc-GnRH conjugate (at a concentration of 5×10^−6^ M) for various time periods (5, 10, 20, 40, 80, 120 and 180 min) before the cellular uptakes were measured at different incubation time points as described above to determine the cellular uptake curves.

### Phototoxicity and Dark Toxicity of ZnPc-GnRH Conjugate

Cells at a density of 2.5×10^5^ per ml in a volume of 200 µl per well were placed in 96-multiwell black opaque plates and incubated overnight at 37°C under 5% CO_2_. One column of wells did not receive cells to serve as a blank. The cells were incubated with ZnPc-GnRH conjugate at various concentrations (10^−7^, 10^−6.5^, 10^−6^, 10^−5.5^, 10^−5^ and 10^−4.5^ M) for 12 hr. One control column in the plate was filled with conjugate-free culture medium. The cells were then washed twice with sterile PBS before fresh medium was added. The plates were illuminated at a light dosage of 1.5 J cm^−2^ using a 670 nm SAS-DL3 medical laser (Beijing Shou’anshan Electronic Technology Co. Ltd. Beijing, China) and then returned to incubator; dark toxicity was measured in parallel. After 24 hr incubation, cell viability was measured on a microplate reader (Synergy 4, BioTek Instruments) using the AlamarBlue® fluorometric cell viability assay (BioSource International Inc., Camarillo, CA, USA) with λ_ex_ 530 nm and λ_em_ 585 nm. The average fluorescence reading of the blank wells was subtracted from the readings of the wells containing cells. Four replicates in each drug dosage were run for each cell line, and each experiment was repeated four times. The cell phototoxicity curves were plotted as a function of drug dose, and IC_50_ values were calculated.

### Statistics

Statistical analysis was performed using GraphPad Prism, version 4.0 (GraphPad Software Inc. San Diego, CA). Quantitative data were expressed as mean ± standard error (SE) from at least three independent experiments and assessed using a one-way ANOVA and a Newman-Keuls test. A probability value of p<0.05 was considered statistically significant.

## Results

### Synthesis and Characterization of ZnPc-GnRH Conjugate

**Figure 1:**
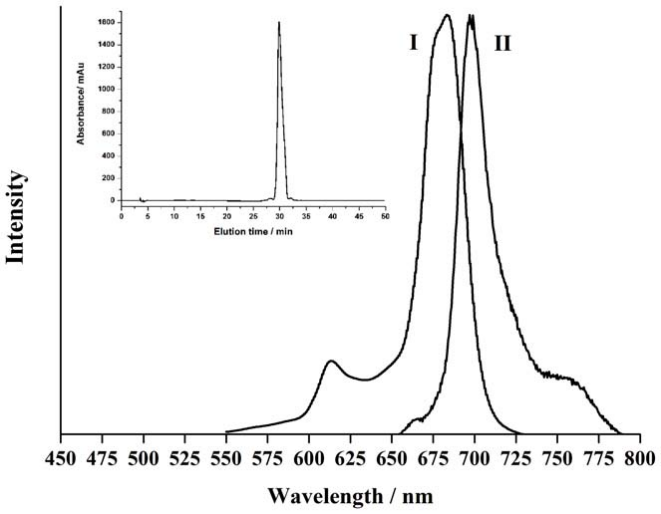
Absorption (I) and fluorescence emission (II, excitation wavelength  = 608 nm) spectra of ZnPc-GnRH (0.5 µM) in DMSO. The inset is the C_18_ reversed-phase HPLC chromatogram.

ZnPc-GnRH conjugate (**7**) was prepared by coupling mono-substituted β-carboxyphthalocyanine zinc (ZnPc-COOH, **5**) with side chain protected GnRH peptide on Wang resin (**Scheme S1**). The conjugate was then de-protected and cleaved from the resin and purified by reversed-phase HPLC to a purity of 99.3% judged by the absorption of ZnPc at 670 nm (**Fig. 1** inset). The structure of ZnPc-GnRH conjugate was confirmed by ESI-MS (**Fig. S1**), ^1^H-NMR (**Fig. S2**) and FT-IR (**Fig. S3**).

The UV/Vis absorption spectrum of ZnPc-GnRH conjugate in DMSO (I in **Fig. 1**) showed a maximum absorption at 684 nm and a shoulder peak at 677 nm. The extinction coefficient was determined to be 312880 L mol^−1^ cm^−1^ for the 684 nm peak, in agreement with previous ZnPc conjugates [8], [19], [22]. The fluorescence emission spectrum of ZnPc-GnRH conjugate in DMSO was also measured (II in **Fig. 1**) and showed a maximal emission peak at 696 nm with excitation at 608 nm. ZnPc-GnRH conjugate can also be excited at 360 nm but with an emission peak height of only 10% of the peak with excitation at 608 nm.

**Figure 2:**
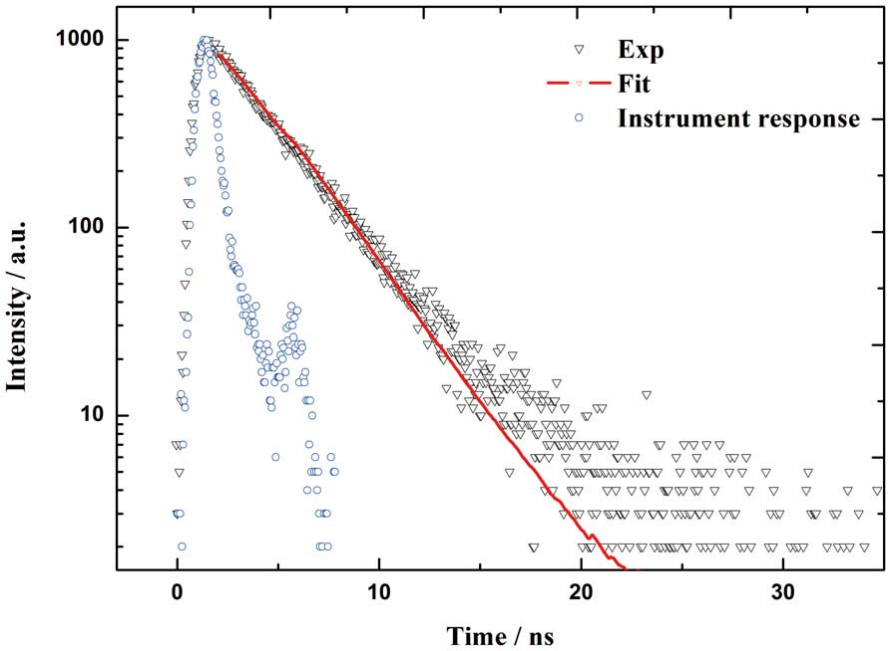
Room temperature fluorescence decays (∇) (excitation wavelength  = 397 nm) and the fitted curve (–○–) of ZnPc-GnRH in DMSO.

We also measured some photophysical parameters of ZnPc-GnRH conjugate in DMSO, which includes the fluorescence quantum yield (Ф_f_) and fluorescence decay time (τ_s_). τ_s_ value of ZnPc-GnRH conjugate was determined based on data-fitting from the fluorescence decay curve (**Fig. 2**). As shown in **Table 1**, Ф_f_ (0.17) and τ_s_ (2.7 ns) of ZnPc-GnRH conjugate were comparable to that of ZnPc reported previously [20], [23], [24]. Meanwhile, the singlet oxygen quantum yield (Ф_Δ_) of ZnPc-GnRH conjugate in DMSO was determined to be 0.73, which is similar to that of ZnPc (0.67 [21]). All above results indicate that the ligation of the GnRH peptide with ZnPc photosensitizer does not significantly alter the photophysical properties of the photosensitizer.

**Table 1:** Photophysical parameters of ZnPc-GnRH conjugate in DMSO compared to that of ZnPc in DMSO.

	Ф_f_	τ_s_ (ns)	Ф_Δ_
ZnPc	0.20 [20]	1.22 [23], 3.6 [24]	0.67 [21]
ZnPc-GnRH	0.17	2.7	0.73

### Cellular Uptake of GnRH Conjugate

**Figure 3:**
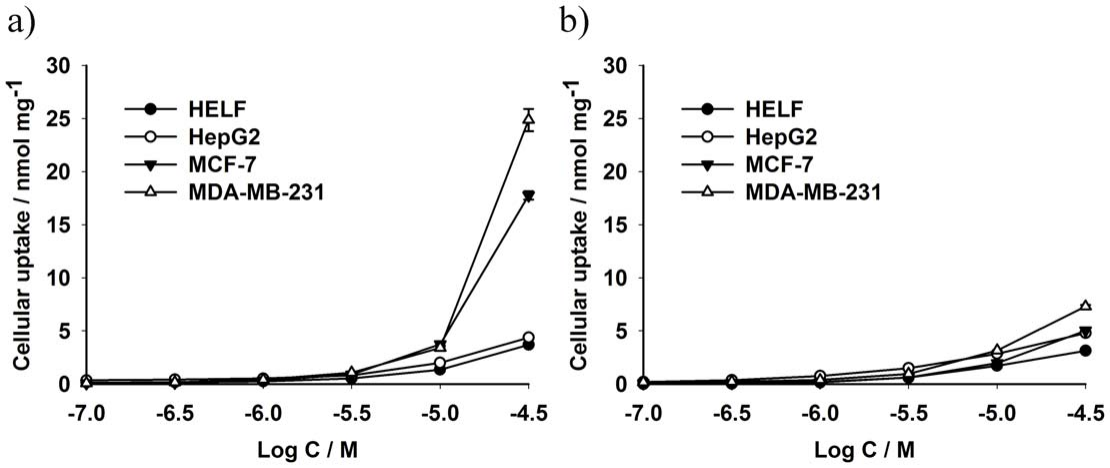
Cellular uptakes of ZnPc-GnRH conjugate (a) and free-GnRH photosensitizer ZnPc-COOH (b) after 12 hr incubation with various cell lines as follows: HELF (•), HepG2 (○), MCF-7 (▾) and MDA-MB-231 (△). Values represent the mean of four independent experiments; bars represent standard error of the mean (SEM).

We chose four cell lines with different levels of GnRH surface receptors to measure the cellular uptakes of ZnPc-GnRH conjugate into these cells. The human breast cancer cell lines (MCF-7 and MDA-MB-231) with over-expressed GnRH receptors [25], [26], [27], HepG2 (human hepatoblastoma cells) with a low level of GnRH surface receptors [28] and HELF (human embryo lung fibroblasts), a cell line with no expression of GnRH receptor [29] were used in this study. All these cells are adherent cells. We incubated these cells with photosensitizer ZnPc-GnRH or GnRH-free conjugate ZnPc-COOH (as a control for the experiment) for 12 hr along with 1% DMSO (for ZnPc-GnRH) or 0.2% Cremophor EL (for ZnPc-COOH) that can facilitate the dissolution of these photosensitizers. The amount of absorbed photosensitizers inside the cells were measured based on their fluorescence signals after the unbound photosensitizers around being washed out. We observed that the cellular uptakes of both photosensitizers (ZnPc-GnRH and ZnPc-COOH) were increased with the increasing amount of photosensitizers applied in all four cell lines above, suggesting that their cellular uptakes are in dose-dependent manners (**Fig. 3**).

ZnPc-GnRH conjugate exhibited significantly higher levels of cellular uptakes in breast cancer cell lines (MCF-7 and MDA-MB-231) as compared to the other two cell lines (HELF and HepG2) (**Fig. 3a**), especially at a higher concentration (10^−4.5^ M). In contrast, the unconjugated photosensitizer (ZnPc-COOH) showed low levels of cellular uptakes by all these four cell lines (**Fig. 3b**). The significantly enhanced cellular uptake of ZnPc-GnRH in breast cancer cells (MCF-7 and MDA-MB-231) over HepG2 or HELF is likely due to the interaction of ZnPc-GnRH conjugate with GnRH receptors expressed on the surface of breast cancer cells.

### Reduced ZnPc-GnRH Accumulation on MCF-7 Cells in the Presence of a GnRH Antagonist

**Figure 4:**
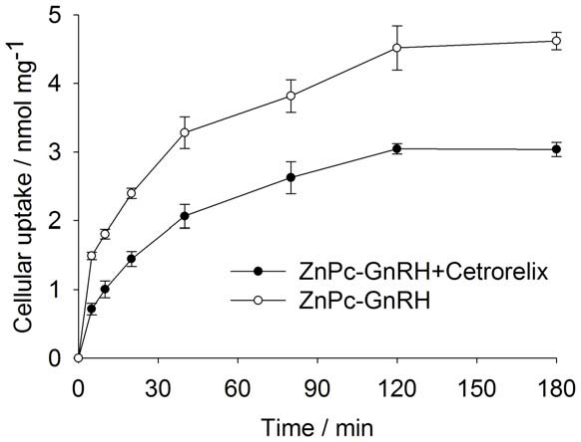
Time course for accumulation of ZnPc-GnRH conjugate in MCF-7 cells at different incubation time points in the presence (○) or absence (•) of pretreatment with GnRH antagonist Cetrorelix (7.5×10^−7^ M, 12 hr). Values represent the mean of four independent experiments; bars represent standard error of the mean (SEM).

To further confirm the selectivity of ZnPc-GnRH conjugate towards breast cancer cells is mediated by the GnRH surface receptor, MCF-7 cells were pre-treated with a GnRH antagonist Cetrorelix (at 7.5×10^−7^ M) for 12 hr, followed by the monitoring cellular uptake of ZnPc-GnRH (at 5.0×10^−6^ M) for 2 hr. If cellular uptake is receptor-mediated, Cetrorelix would be expected to compete with ZnPc-GnRH for the same binding sites on the cell membrane and reduce the binding and the internalization of ZnPc-GnRH conjugate. Indeed, this proved to be the case (**Fig. 4** **)**. In this particular experiment, Cetrorelix did not completely displace the uptake of ZnPc-GnRH probably due to the relatively low amount of Cetrorelix used. It was difficult to use higher amount of Cetrorelix in the experiment because Cetrorelix peptide at a higher concentration interferes with ZnPc fluorescence, which was the readout of the amount of ZnPc absorbed by cells.

It was showed in **Fig. 4** that the cellular uptake of ZnPc-GnRH increased over time and reached the maximal level at approximate 2 hr. This time dependence is consistent with that observed in a previous experiment using a different GnRH conjugate (GnRH-protoporphyrin IX [30]).

### Phototoxicity and Dark Toxicity of GnRH Conjugate

**Figure 5:**
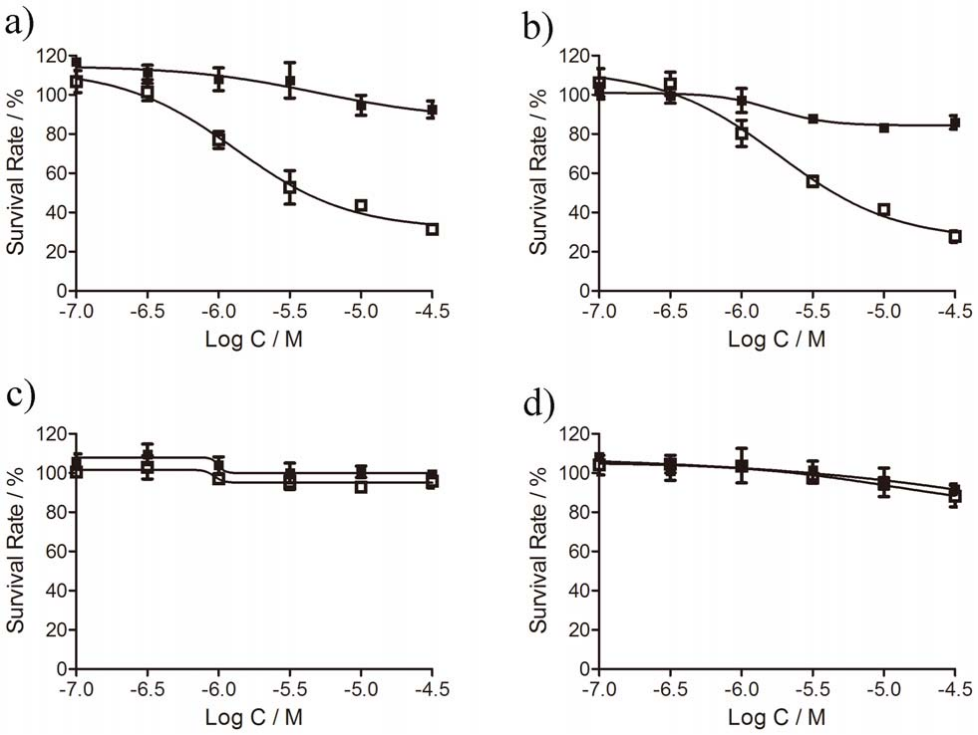
Phototoxicity (□) (at a light dosage of 1.5 Jcm^−2^) and dark toxicity (▪) of ZnPc-GnRH conjugate at various concentrations after 12 hr incubation with MDA-MB-231 (a), MCF-7 (b), HELF (c), and HepG2 (d). Values represent the mean of four independent experiments; bars represent standard error of the mean (SEM).

We measured both phototoxicity and dark toxicity of ZnPc-GnRH on the four cell lines (MDA-Mb-231, MCF-7, HELF and HepG2). Consistent with the results we obtained from cellular uptake experiments, ZnPc-GnRH exhibited remarkable dose-dependent phototoxicities (□) in both breast cancer cell lines (MCF-7 and MDA-MB-231, as shown in **Fig. 5a and 5b**) after 12 hr incubation. The concentrations required to yield 50% growth inhibition (IC_50_) were 1.32 µM for MDA-MB-231 and 1.82 µM for MCF-7, respectively. In contrast, no significant phototoxicity was found in HELF (**Fig. 5c**) or HepG2 (**Fig. 5d**) after 12 hr incubation with ZnPc-GnRH. In addition, no obvious cellular dark toxicity (▪) of ZnPc-GnRH was observed among these four cell lines, suggesting safety of ZnPc-GnRH in the absence of light. Putting together, all these results suggest that ZnPc-GnRH is a highly selective tumor-targeting photosensitizer.

## Discussion

The photosensitizer is one of the three key factors that play critical roles in PDT. Phthalocyanine-based photosensitizers have attracted increasing attentions for their advantageous properties compared to the most widely used porphyrin derivatives. Phthalocyanine has a maximal absorbance around 670 nm, at which the depth of light penetration in tissue is twice that of porphyrin at 630 nm [31]. Moreover, phthalocyanine has much less absorption at solar radiation spectrum (maximum intensity at 475 nm) compared to porphyrin [32], which may lower skin phototoxicity.

A number of phthalocyanine-based photosensitizers are now in clinical applications [33], [34] or under clinical trials [35]. None of them is a single component compound. Rather, all of them are mixtures of different phthalocyanine isomers. Such chemical heterogeneity is the results from the typical statistical condensation synthetic method for derivatized phthalocyanine. Recently, there is an increasing interest in synthesizing phthalocyanine-based photosensitizers with single component, which facilitate the detailed study of structure-function relationship and is also preferred by drug regulatory agencies. Van Lier used Kobayashi subphthalocyanine ring expansion reaction to synthesize 3∶1 asymmetrically substituted dodecafluorinated phthalocyanine, which exhibited an improved photodynamic activity compared to the symmetric hexadecafluorophthalocyanine [36]. Dennis Ng group has made significant contributions in developing synthetic methods for asymmetric phthalocyanines without isomers. In their work, phthalocyanine was derivatized and connected with glycans [37], polyethylene glycols [38] or 1,3-bis(dimethylamino)-2-propoxy group [39] to enhance the hydrophilicity of phthalocyanine and the selectivity for tumor cells. We previously developed a brute approach to generate pure ZnPc-COOH compound in large quantity [19]. The carboxy group in ZnPc-COOH allows the ligation of different targeting agents onto ZnPc-COOH. In this study, we ligate GnRH peptide to ZnPc-COOH with solid-phase synthesis, through which many sorts of peptides or oligonucleotides can be connected with phthalocyanine zinc derivatives.

Besides GnRH analogues, a number of bio-molecules have been used to guide photosensitizers to tumor cells. Maleylated bovine serum albumin was conjugated to phthalocyanine and showed promising results with increased cellular uptakes through macrophage scavenger receptor [40]. Similarly, the conjugation of AlPcS4 with monoclonal anti-carcinoembryonic-antigen antibody was reported to facilitate the cellular uptake [41]. Nevertheless, conjugates of peptides to photosensitizers are preferable due to a number of reasons. Compared to the other bio-molecules, peptides are usually readily accessible by chemical synthesis and can have well-defined chemical linkages to photosensitizer. Besides, peptide could be cleared out quickly, leading to lower background for imaging purpose. Stefflova *et al*. conjugated a fluorescent photosensitizer (Pyropheophorbide A) to a quencher through a peptide linker (GDEVDGSGK) that can be cleaved by caspase-3, a key caspase involved in apoptosis [42]. This conjugate functioned not only as a photosensitizer that leads to photodynamic effect, but also as a near-infrared imaging agent to detect cellular apoptosis accompanying photodynamic effect. The fluorescence of Pyropheophorbide A was quenched by the quencher in the conjugate. In the events of apoptosis, active caspase-3 cleaved the peptide linker, thus removed the quencher and led to the fluorescence signal. Poly-arginine was also used to conjugate to a photosensitizer (Tetraphenylchlorin, TPC) to improve the hydrophilicity and cell-penetrability [43]. It was observed that the cellular uptake of this conjugate was enhanced about 8 times than that of the unbound photosensitizer. Tirand and his colleagues reported a conjugate which consisted of TPC, aminocaproic acid (Ahx) and peptide. The peptide ATWLPPR, which was identified from phage display screening, can specifically bind to one of the vascular endothelial growth factor (VEGF) receptors. Compared to unmodified TPC, the conjugate showed almost 10 times higher phototoxicity toward human endothelial cell [44], indicating the potential photodynamic activity through peptide-mediated VEGF receptor targeting.

In our case, ligation with GnRH analogue does not perturb the photophysical properties of the parent ZnPc, meanwhile, improves the ZnPc’s solubility in aqueous circumstance. ZnPc-GnRH conjugate was found to accumulate preferentially on the cells expressing a high level of GnRH receptor compared to the cells with a low expression of GnRH receptor, or compared to photosensitizer without the targeting peptide (ZnPc-COOH). The phototoxicity of ZnPc-GnRH conjugate is found specific to GnRH-receptor-expressing cells.

Furthermore, the UV/Vis absorption spectrum of ZnPc-GnRH conjugate in DMSO (I in **Fig. 1**) showed a maximum absorption at 684 nm and a shoulder peak at 677 nm. For comparison, the maximal absorption (in DMSO) is at 670 nm for ZnPc, 674 nm for ZnPc-COOH, 678 nm for pentalysine phthalocyanine zinc (ZnPc-(Lys)_5_), and 670 nm for di-sulfonated di-phthalimidomethyl phthalocyanine zinc (ZnPc-S_2_P_2_) [45]. The absorption peak at 684 nm may represent a population of ZnPc-GnRH molecules where GnRH peptide extends to the top of the phthalocyanine ring and binds to the central zinc atom at axial position. Such intramolecular interaction may then affect the UV absorption peak in a way resemble the solvent effect on ZnPc absorption [45].

In conclusion, we conjugated a photosensitizer (ZnPc) to a hormone peptide, GnRH, which is highly expressed in breast tumors. This ZnPc-GnRH conjugate was prepared to a single component with a high purity. The cellular uptake and phototoxicity of the ZnPc-GnRH conjugate were examined with several cell lines on which different levels of GnRH receptors are expressed. The cellular uptakes of ZnPc-GnRH conjugate were found in a dose- and time- dependent manner in all cells, but the uptakes are much higher in tumor cells than in cells with low levels of GnRH receptor. The uptake of this ZnPc-GnRH conjugate can be competed off by a specific GnRH receptor blocker (Cetrorelix), further supporting that this conjugate is specific to GnRH receptors expressed on the tumor cell surface. We also demonstrate the selective phototoxicity toward cells expressing GnRH. This study thus paves the road for further PDT efficacy study of GnRH conjugates in animals.

## Supporting Information

Figure S1 **Mass spectrum of ZnPc-GnRH by ESI HRMS (DECAX-30000 LCQ Deca XP).** *m/z* peaks at 1806.1 and 903.5 correspond to the [M+H]^+^ and [M+2H]^2+^ ions.
(TIF)

Figure S2 **Proton nuclear magnetic resonance (^1^H-NMR, Bruker AV-400, 400 MHz, [D_6_]DMSO) of ZnPc-GnRH measured in deuterated DMSO and the tentative assignment of the chemical shifts.**
(TIF)

Figure S3 **Infrared spectrum of ZnPc-GnRH.** The spectrum was determined by FT-IR Spectrometer (Magna-IR 750, Nicolett, KBr). υ□ = 3271 (N-H stretch), 1653 (amide I), 1530 (amide II), 1202 (COO^–^ stretch) cm^–1^.
(TIF)

Scheme S1 **Synthesis of ZnPc-GnRH conjugate (7) from the previously described monosubstituted β-carboxyphthalocyanine zinc (5, ZnPc-COOH).** Reagents and conditions: a) (NH_2_)_2_CO, Zn(OAc)_2_, (NH_4_)_2_MoO_4_, 170°C, 4 hr; b) KOH (1 M), 100°C, 24 hr; c) HBTU, DIEA, DMF, 25°C, 24 hr; d) TFA (95%), 25°C, 4 hr.
(TIF)
