# Supplementary figures and images for: Receptor-Targeting Phthalocyanine Photosensitizer for Improving Antitumor Photocytotoxicity
Source: PLoS One. 2012 May 31;7(5):e37051. doi: 10.1371/journal.pone.0037051 (PMC3365043; doi:10.1371/journal.pone.0037051)

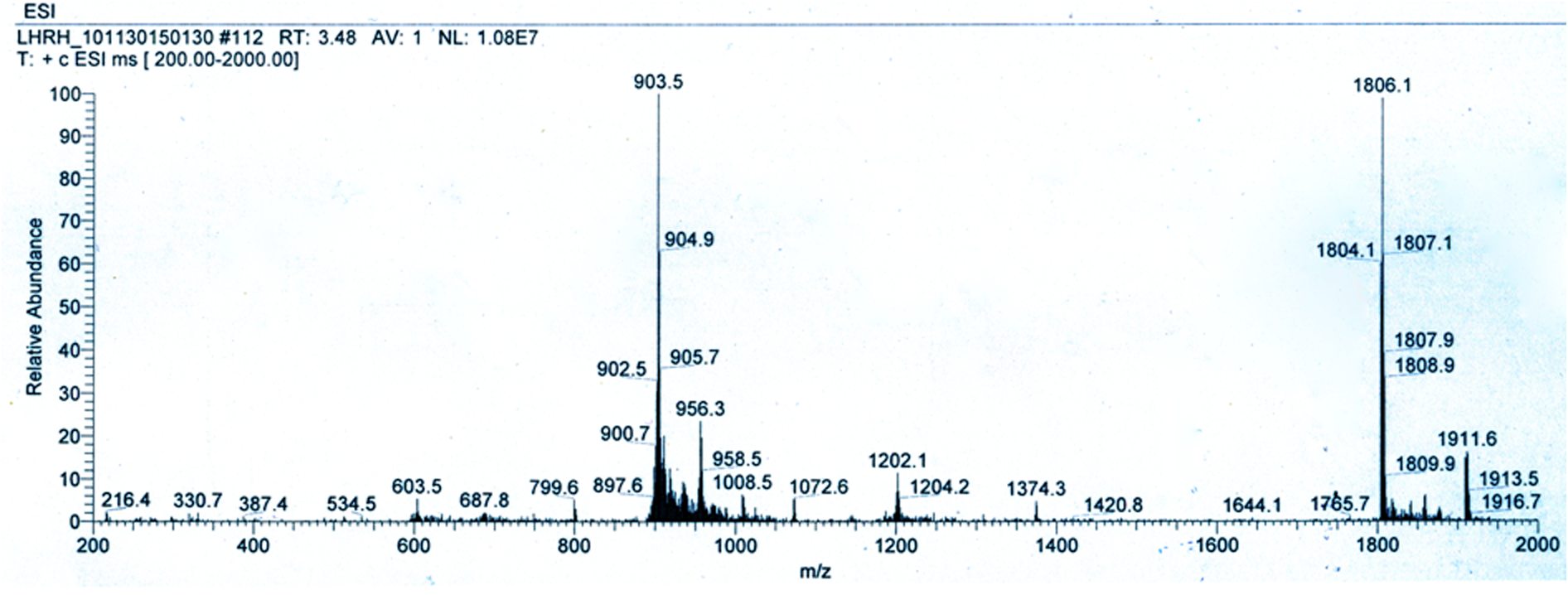

Supplement: Figure S1 — Mass spectrum of ZnPc-GnRH by ESI HRMS (DECAX-30000 LCQ Deca XP). m/z peaks at 1806.1 and 903.5 correspond to the [M+H]+ and [M+2H]2+ ions. (TIF) [file pone.0037051.s001.tif]

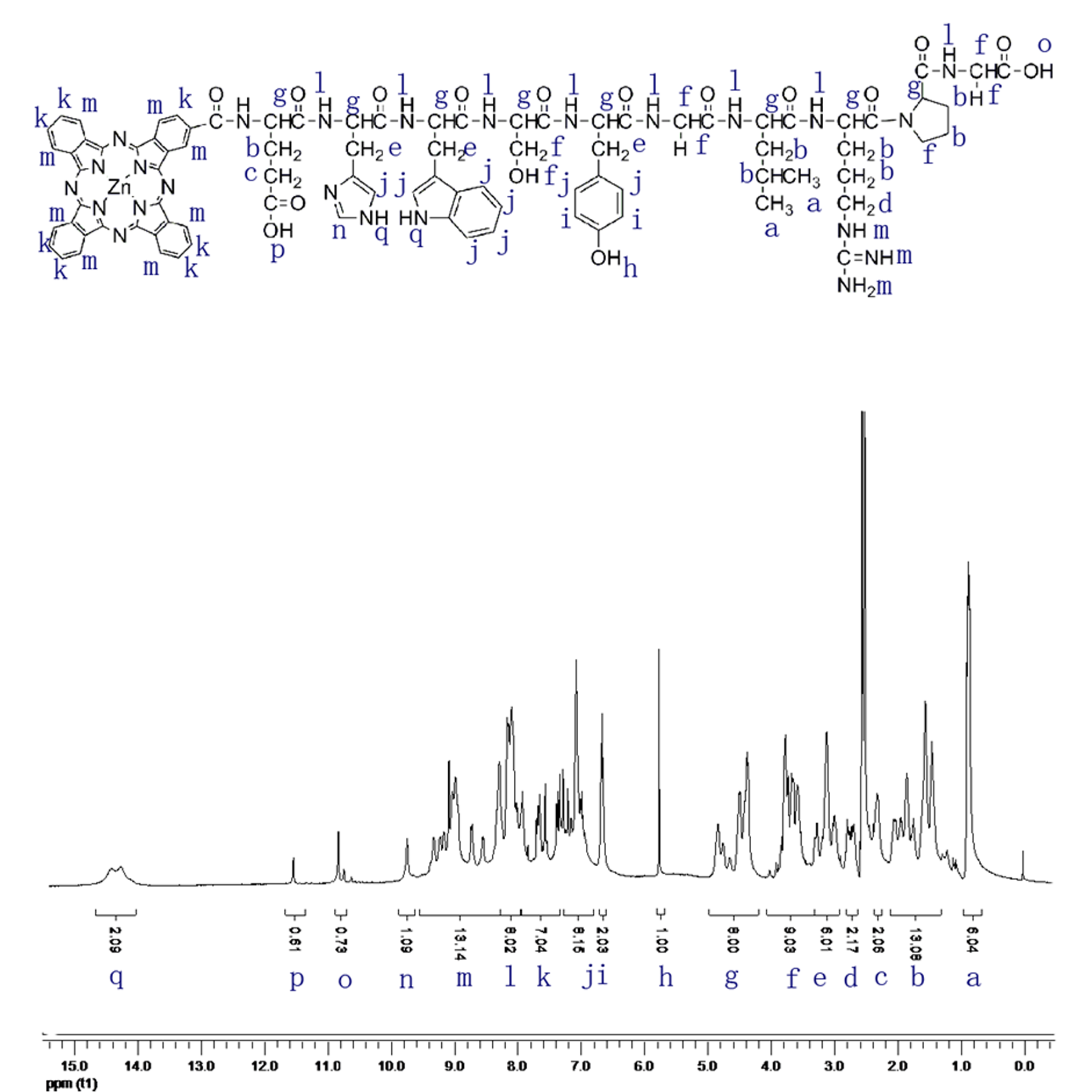

Supplement: Figure S2 — Proton nuclear magnetic resonance (1H-NMR, Bruker AV-400, 400 MHz, [D6]DMSO) of ZnPc-GnRH measured in deuterated DMSO and the tentative assignment of the chemical shifts. (TIF) [file pone.0037051.s002.tif]

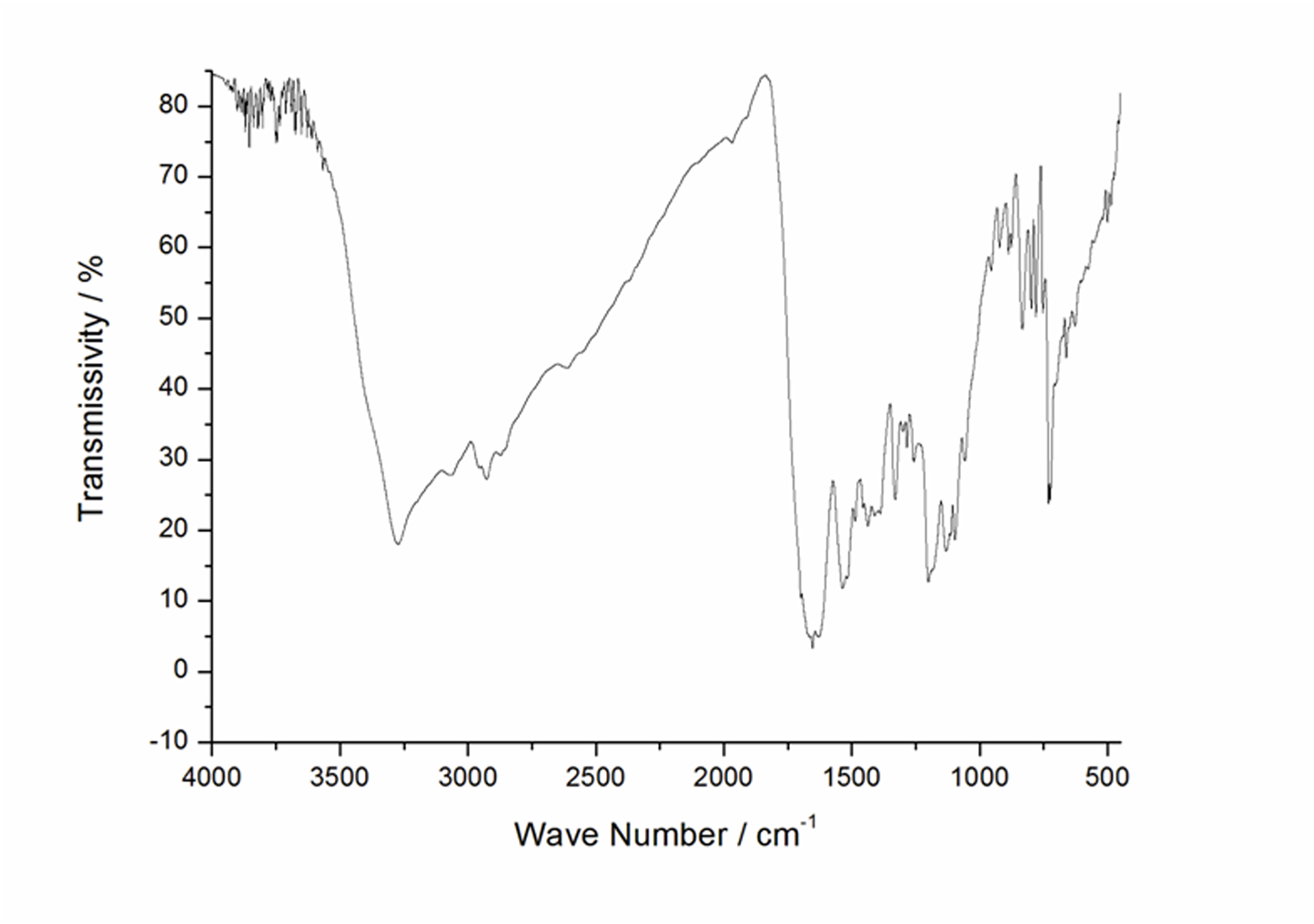

Supplement: Figure S3 — Infrared spectrum of ZnPc-GnRH. The spectrum was determined by FT-IR Spectrometer (Magna-IR 750, Nicolett, KBr). υ□ = 3271 (N-H stretch), 1653 (amide I), 1530 (amide II), 1202 (COO– stretch) cm–1. (TIF) [file pone.0037051.s003.tif]

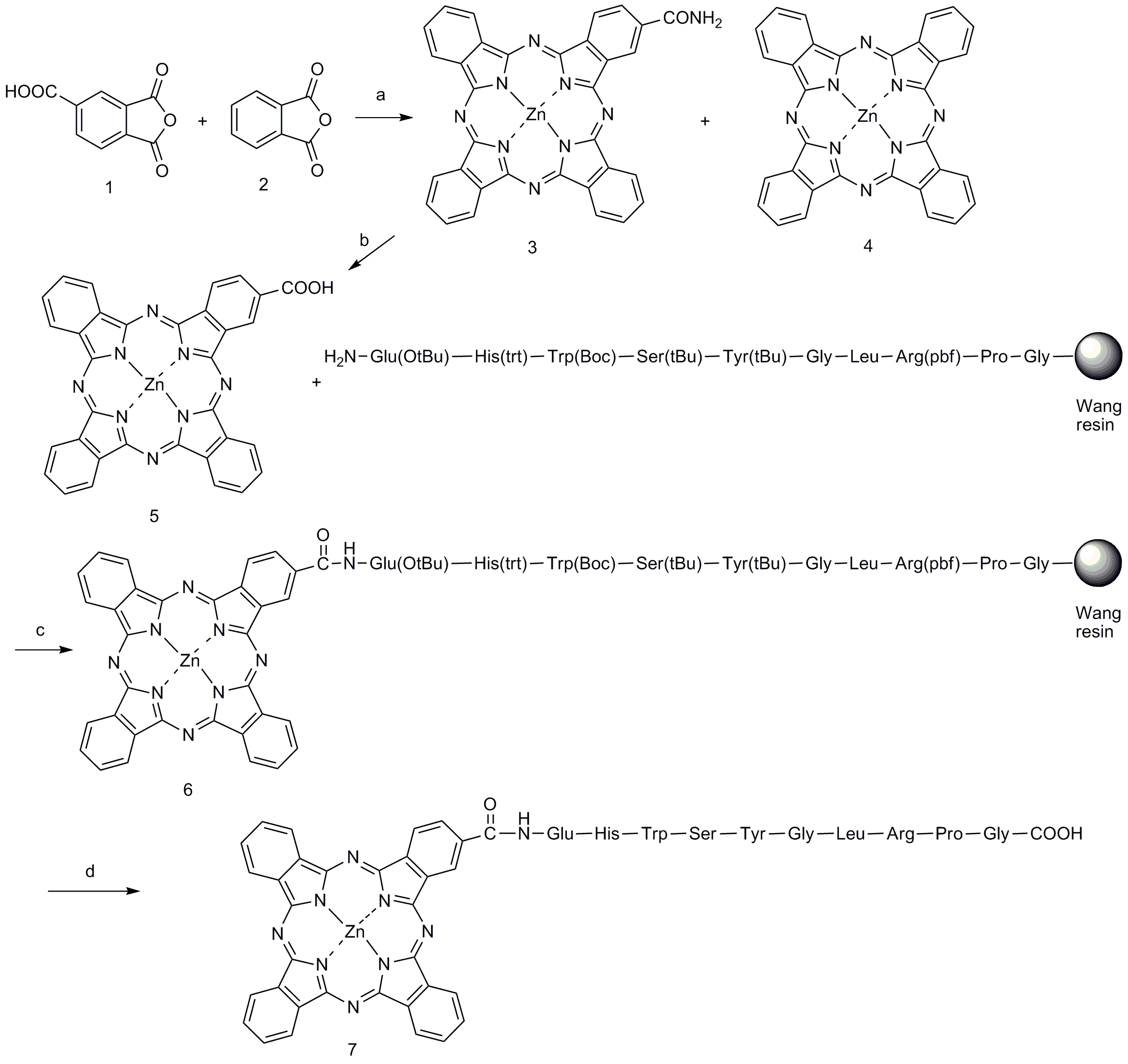

Supplement: Scheme S1 — Synthesis of ZnPc-GnRH conjugate (7) from the previously described monosubstituted β-carboxyphthalocyanine zinc (5, ZnPc-COOH). Reagents and conditions: a) (NH2)2CO, Zn(OAc)2, (NH4)2MoO4, 170°C, 4 hr; b) KOH (1 M), 100°C, 24 hr; c) HBTU, DIEA, DMF, 25°C, 24 hr; d) TFA (95%), 25°C, 4 hr. (TIF) [file pone.0037051.s004.tif]
